# Early weaning causes small intestinal atrophy by inhibiting the activity of intestinal stem cells: involvement of Wnt/β-catenin signaling

**DOI:** 10.1186/s13287-023-03293-9

**Published:** 2023-04-05

**Authors:** Junquan Tian, Yuying Li, Xuetai Bao, Fan Yang, Xiongzhuo Tang, Qian Jiang, Yulong Yin, Kang Yao

**Affiliations:** 1 National Engineering Laboratory for Pollution Control and Waste Utilization in Livestock and Poultry Production, Key Laboratory of Agro-Ecological Processes in Subtropical Region, Laboratory of Animal Nutritional Physiology and Metabolic Process, Institute of Subtropical Agriculture, Chinese Academy of Sciences, Changsha, 410125 Hunan China; 2 University of Chinese Academy of Sciences, Beijing, 100008 China; 3 College of Animal Science and Technology, Hunan Agricultural University, Changsha, 410000 Hunan China

**Keywords:** Early weaning, Intestinal stem cells, Wnt/β-catenin, Intestinal growth, Epithelial regeneration

## Abstract

**Background:**

Early weaning and shorter breastfeeding duration are applied by a proportion of young mothers, especially in the social spheres of poverty-stricken areas. Early childhood is a critical period for intestinal development, which is driven by intestinal stem cells (ISCs). However, how early weaning practice affects the function of ISCs to mediate intestinal development remains unclear.

**Methods:**

We established an excellent early weaning mice model that has significant intestinal atrophy and growth arrest symptoms to explore the responses of ISCs to early weaning. The primary and passaged intestinal organoids from the suckling or early weaning mice were cultured to explore the underlying mechanism of early weaning affecting the ISCs.

**Results:**

Early weaning depressed the self-renewal of ISCs and attenuated the activity of ISCs-driven intestinal epithelial regeneration and crypt expansion in vivo and ex-vivo. Further results showed that early weaning retarded the differentiation of ISCs into transit-amplifying cells and Paneth cells, and accelerated the apoptosis of villous epithelial cells, jointly leading to intestinal epithelial atrophy. Mechanistically, early weaning inhibited Wnt signaling in ISCs, while an exogenous Wnt amplifier restored ISCs’ function in ex-vivo.

**Conclusion:**

Our findings indicate that early weaning depresses the activity of ISCs via attenuating Wnt/β-catenin signaling and triggers the proinflammatory cytokines TNF-α, IL-1β, IL-6, and IL-17 in jejunum, thereby impeding ISCs-driven epithelial regeneration and intestinal growth, which may provide a basal theory for the development of infant nutrients targeting stem cells to alleviate early weaning-induced intestinal problems.

**Supplementary Information:**

The online version contains supplementary material available at 10.1186/s13287-023-03293-9.

## Introduction

World health organization (WHO) recommended that infants should be breastfed exclusively during the first six months of life, and furtherly be breastfed together with supplementary feeding until the baby reaches two years of age [1, 2]. In practice, a proportion of young mothers have to apply earlier weaning to their babies, due to insufficient breast milk, social stress, and work burdens [2–4]. Studies have evidenced that this early weaning practice may increase the incidence of gastrointestinal disease [5–7]. For the piglets, early weaning usually leads to small intestinal morphological abnormality and dysfunction in absorption and barrier [8]. As a result, early weaning piglets commonly experience poor growth performance and increases in diarrhea and mortality [9]. Although early weaning-induced intestinal atrophy has been widely reported in mammals, the involved mechanism is still incompletely understood.

The small intestine is one of the most rapidly renewing tissues, regenerating every 2 to 4 days in mammals [10, 11]. Small intestinal development is driven by ISCs [12]. In general, ISCs, lying in the crypt regions, divide asymmetrically into a stem cell and a transit-amplifying cell. The latter differentiate into multiple functional epithelial cells, including enterocytes, Paneth cells, goblet cells, enteroendocrine cells, and tuff cells [12, 13]. Except for Paneth cells that maintain in the crypt, these functional epithelial cells migrate from crypts to villi, undergo programmed cell death, and drop from the villi [14, 15]. Besides, recently accumulated evidence suggests ISCs play a critical role in epithelial regeneration from damage. For instance, one previous study found that rotavirus infection accelerates the regeneration of small intestinal epithelium by activating ISCs, to replace the infected lost cells [16]. In contrast, some circumstances, including inflammation, endoplasmic reticulum stress, aging, and chemotherapy, will attenuate the activity of ISCs, which increases the difficulty of the intestine recovering from the epithelial damages [17–20]. Interestingly, a recent study revealed that early life stress will disrupt intestinal epithelium homeostasis in adulthood by regulating the self-renewal and differentiation of ISCs [21]. Therefore, it can be inferred that a better understanding of how early weaning affects the function of ISCs may provide novel intervention strategies for alleviating early weaning-induced intestinal atrophy. Given that Wnt signaling in the ISCs is a critical pathway for the epithelial regeneration and growth of the small intestine [16, 22, 23], we hypothesize that the Wnt signaling pathway in ISCs is involved in early weaning-induced intestinal epithelial atrophy.

In the present study, for the first time, we established an excellent early weaning mice model, and discovered that early weaning attenuates the function of ISCs to drive intestinal development in vivo and ex-vivo by inhibiting Wnt/β-catenin signaling. These findings reveal the mechanism underlying early weaning-induced small intestinal atrophy from the perspective of ISCs, which may provide an additional basal theory for the development of infant nutrients to alleviate early weaning-induced intestinal health problems.

## Materials and methods

### Animals

All animal experiments were performed in accordance with the guidelines of the Laboratory Animal Ethical Commission of the Institute of Subtropical Agriculture, the Chinese Academy of Sciences.

Pregnant female wild-type C57BL/6 mice were purchased from the SLAC Laboratory Animal Central (Changsha, China). All pregnant mice and their pups were housed in a pathogen-free mouse colony, kept on a 12 h light/dark cycle, a constant ambient temperature of 25 ± 2 °C, relative humidity of 45–60%, fed with standard laboratory chow, and applied with water ad libitum. A total of 93 mice were used in our study, including 8 adult female mice, 3 adult male mice, and 82 of their pups. We declare that our study adheres to the ARRIVE guidelines for the reporting of animal experiments.

### Animal experiments design

All pups were weaned on postnatal day 15. On postnatal day 14, the mother was taken away from their pup’s cage for 18 h consecutively (3:00 pm–9:00 am). After separation, the mother was returned to their pup’s cage for 6 h (9:00 am–3:00 pm), which is to make the pup better to adapt the life after weaning, and the pups in the weaned group were taken away from their mother forever. To investigate the effects of early weaning on pups, early weaning pups from the same litter were randomly assigned into 2 groups based on the rule of similar body weights. The mice in the weaned group lived alone and were supplemented with a solid diet which was formulated according to previous studies [24], while the mice in the suckling group lived with their mother. The body weights were monitored every day. Total food intake of the weaned pups was recorded on day 7 of post-weaning. On day 3 or 7 post-weaning, the mice were sacrificed to collect jejunum tissues for further analysis. To determine cell proliferation and migration in the intestinal epithelium, mice were intraperitoneally injected with 5-ethynyl-2′-deoxyuridine (EdU) (25 mg/kg in phosphate buffer saline, PBS) at 24 h before harvest.

### Enteroids culture

Enteroid culture was conducted as described previously with some modifications [12]. Briefly, approximately 5 cm of jejunum were harvested from mice sacrificed by cervical dislocation on day 3 post-weaning and flushed with ice-cold PBS. The jejunum segments were opened by the longitudinal incision, followed by removing the villi. Next, the jejunum segment was cut into 2-mm fragments. Then, these fragments were shaken with a solution of 2.5 mM EDTA (Invitrogen™) in PBS for 15 min on the ice. To isolate crypts, the fragments were thoroughly suspended by pipetting up and down by 15 times with a fetal bovine serum rinsed 10 mL tip (FBS, Gibco). To remove other tissue blocks, suspensions with crypts were filtered with a 70-μm cell strainer (Corning). To remove single cells, crypt suspension was supplemented with 10% (vol/vol) FBS and spun down at 300 × *g* at 4 °C for 3 min. Next, the supernatant was discarded, while the pellet was re-suspended in 10 mL DMEM/F12 media (Gibco™), and then crypts were collected by spinning down at 100×*g* at 4 °C for 2 min. Approximately, 300 crypts were suspended in a 50 μL matrix composed of 25 μL DMEM/F12 medium (Gibco™) and 25 μL growth factor reduced phenol-red free Matrigel (Corning, Bedford, USA), and seeded in the center of each well of a 24-well plate. After matrigel solidification, 500 μL enteroid standard medium containing DMEM/F12 Gln-free medium (Gibco™), 2 mM glutamine (Gibco™), 10 mM HEPES (Gibco™), B27 supplement (Invitrogen), N2 supplement (R&D System), recombinant murine epidermal growth factor (EGF, 50 ng/mL, Pepro Tech), recombinant mouse R-spondin1 (500 ng/mL, R&D system), and recombinant mouse Noggin (100 ng/mL, Pepro Tech) was added. The passage was performed every 3–4 days with a 1:3 split ratio. The images of organoids were taken using an optical microscope (Nikon, TE2000, Japan) and the acquisition software is NIS-Elements. The resolution for exported images is 300 dots per inch (DPI).

### Immunofluorescence for enteroids

To harvest intact enteroids, matrigel was dissolved using iced PBS. Enteroids suspension was centrifuged with 100×*g* for 5 min. To fix enteroids, the precipitation was resuspended softly with 4% paraformaldehyde at room temperature for 10 min. Then, the fixed enteroids were spun down at 500×*g* at 4 °C for 5 min using the CytoSpin Cytocentrifuge (Yingtai Ltd, Changsha, China) to attach on slides (Thermo Scientific™ Superfrost™ Plus). The enteroids on the slides were circled with a PAP PEN, followed by washing with PBS for 3 × 2 min. Then, the slides were treated with 5% (vol/vol) Triton-X 100. After 5 min, the slides were washed with PBS for 3 × 2 min and incubated with 5% defatted milk solution to block background staining for 1 h. Subsequently, the slides were incubated with primary antibodies Olfm4 (Rabbit, Cell Signaling Technology, 1:400), β-catenin (Rabbit, Cell Signaling Technology, 1:100), and Ki67 (Rabbit, Cell Signaling Technology, 1:50) in a humidity chamber overnight at 4 °C. The slides were washed 3 × 2 min with PBST (PBS with 0.05% Tween-20) before the slides were incubated with anti-rabbit IgG (H + L), F (ab′)_2_ fragment secondary antibody (Cell Signaling Technology, 1:1000) for 1 h. Finally, after the nuclei were stained with DAPI (1:1000, Beyotime Biotechnology, Shanghai, China), the slides were sealed with a fluorescence anti-quenching agent (Beyotime Biotechnology, Shanghai, China) and cover slides. Images were taken with a confocal laser scanning microscope (Zeiss, LSM880, Germany) driven by the acquisition software ZEN. The resolution for exported images is 300 DPI.

### Histology and immunofluorescence staining

To analyze villus length and crypt height, hematoxylin and eosin (H&E) staining of jejunum was performed as previously described [25]. Briefly, approximately 3 cm jejunum of mice were collected, fixed in 4% paraformaldehyde, washed with PBS, dehydrated with alcohol, and embedded in paraffin blocks. Then, the sections were deparaffinized, hydrated, and stained with H&E. The images were taken using an optical microscope (Olympus, BX51, Japan) and the acquisition software is OLYMPUS Stream. The resolution for exported images is 300 DPI. Villous height and crypt depth were determined by ImageJ software. The crypt fission rate was determined according to the method described in a previous study [26]. To perform immunofluorescence staining, the sections were deparaffinized and followed by antigen retrieval with 10 mM sodium citrate in a 95 °C water bath for 40 min. Then, sections were blocked for 1 h with 5% nonfat milk. Sections of jejunum were incubated with Olfm4 (Rabbit, Cell Signaling Technology, 1:400), Lgr5 (Rabbit, Abcam, 1:100), β-catenin (Rabbit, Cell Signaling Technology, 1:100), Ki67 (Rabbit, Cell Signaling Technology, 1:50), Lysozyme (Rabbit, Abcam, 1:100) or chromogranin A (Rabbit, Santa Cruz, 1:200) primary antibodies overnight at 4 °C. Afterward, the sections were incubated with anti-rabbit IgG (H + L), F (ab′)_2_ fragment secondary antibody (Cell Signaling Technology, 1:1000). The nuclei were stained with DAPI (1:1000, Beyotime Biotechnology, Shanghai, China) for 5 min at room temperature. To detect the migration height of EdU-positive cells, the Click-iT™ EdU Alexa Fluor™ 488 imaging Kit (Invitrogen™) was used according to the manufacturer’s protocols. To detect goblet cells, the PAS staining was performed using the PAS staining kit (Sigma) following the manufacturer’s suggested protocol. For apoptotic cells of villi top detection, terminal deoxynucleotidyl transferase-mediated dUTP nick labeling (TUNEL) was performed by using TUNEL Kit (Keygen Biotech, Nanjing, China) according to the manufacturer’s instructions. The pictures were taken with a confocal laser scanning microscope (Zeiss, LSM880, Germany) driven by the acquisition software ZEN. The resolution for exported images is 300 DPI.

### Real-time quantitative PCR (RT-qPCR)

Total RNA was isolated from jejunum samples using E.Z.N.A.^R^ Total RNA Kit I (Omega Bio-Tek, USA) according to the manufacturer’s instructions. Total RNA from enteroids was extracted using E.Z.N.A.^R^ MicroElute Total RNA Kit (Omega Bio-Tek, USA) according to the manufacturer’s instructions. Total RNA was quantified with Nanodrop ND-1000 (Wilmington, DE, USA) and stored at − 80 °C. cDNA was synthesized using a reverse transcription system from TAKARA (TAKARA BIO). RT-qPCR was performed using TB Green™ (TAKARA BIO) according to the manufacturer’s instructions on a LightCycler480®IImachine. β-Actin was used as an endogenous reference to normalize the quantifies of target mRNA by using the formula 2^−ΔΔCT^(ΔΔCT = ΔCT_sample_-ΔCT_control_). All primer sequences were listed in Table 1.

**Table 1:** Primer sequence of the genes for RT-PCR in the study

Gene	Species	Forward primer 5′–3′	Reverse primer 5′–3′
β-Actin	Mouse	GGCTGTATTCCCCTCCATCG	CCAGTTGGTAACAATGCCATGT
Lgr5	Mouse	CCTACTCGAAGACTTACCCAGT	GCATTGGGGTGAATGATAGCA
Olfm4	Mouse	CAGCCACTTTCCAATTTCACTG	GCTGGACATACTCCTTCACCTTA
Ascl2	Mouse	TTTCCTGTGCCGCACCAGAACT	CAGCGACTCCAGACGAGGTGG
Hopx	Mouse	ACCACGCTGTGCCTCATCGC	TTCTGACCGCCGCCACTCTG
Tert	Mouse	GCACTTTGGTTGCCCAATG	GCACGTTTCTCTCGTTGCG
Msi1	Mouse	GTTCATCGGAGGACTCAGTTGG	CTGGTCCATGAAAGTGACGAAGC
Ephb3	Mouse	CCTGTGTCAAGATCGAGGAGGT	CTTCAGCGTCTTGATAGCCACG
β-catenin	Mouse	GTTACGGCAATCAGGAAAGC	GACAGACAGCACCTTCAGCA
Cmyc	Mouse	TTCATCTGCGATCCTGACGAC	CACTGAGGGGTCAATGCACTC
Cd44	Mouse	CACCATTGCCTCAACTGTGC	TTGTGGGCTCCTGAGTCTGA
Axin2	Mouse	GGACTGGGGAGCCTAAAGGT	AAGGAGGGACTCCATCTACGC
Prom1	Mouse	CTCCCATCAGTGGATAGAGAACT	ATACCCCCTTTTGACGAGGCT
Zfp652	Mouse	GGAGCTGGTTGAACCCTGTG	AGGGCTTCCAGACTCCCTTTT
Vill	Mouse	TTCTACGGTGGTGACTGCTACC	TGGTCCAACAGGACGGCTTGAT
Mmp7	Mouse	CTGCCACTGTCCCAGGAAG	GGGAGAGTTTTCCAGTCATGG
Lysozyme	Mouse	TACAACCGTGGAGACCGAAGCA	TGGCTGCAGTGATGTCATCCTG
Muc2	Mouse	ATGCCCACCTCCTCAAAGAC	GTAGTTTCCGTTGGAACAGTGAA
ChgA	Mouse	AGAACCAGAGCCCTGATGCCAA	CTCTGTGGTTGCCTCAAAGCCA
TNF-α	Mouse	AGGCACTCCCCCAAAAGAT	TGAGGGTCTGGGCCATAGAA
IL-1β	Mouse	ATGAAAGACGGCACACCCAC	GCTTGTGCTCTGCTTGTGAG
IL-6	Mouse	TGCAAGAGACTTCCATCCAGT	GTGAAGTAGGGAAGGCCG
IL-17	Mouse	TACCTCAACCGTTCCACGTC	TTTCCCTCCGCATTGACAC

### Western blot analysis

Western blot analysis was conducted according to our previous study [25]. Briefly, enteroids cytoplasmic and nuclear proteins are extracted using the Nuclear and Cytoplasmic Protein Extraction Kit (Yeasen, Shanghai, China) according to the manufacturer’s instructions. Crypts and tissue total protein were extracted with a strong RIPA Lysis Buffer (Beyotime Biotechnology, Shanghai, China). Protein concentrations were measured by a BCA kit (Beyotime Biotechnology, Shanghai, China) followed by adjusting them to a uniform concentration. The protein samples were mixed with a 5 × SDS-PAGE Sample Loading Buffer (Beyotime Biotechnology, Shanghai, China), followed by boiling at 95 °C for 5 min. Then, the proteins were transferred onto polyvinylidene difluoride (PVDF) membranes and blocked with 5% defatted milk in Tris-Tween saline buffer for 1 h, followed by incubating with corresponding primary antibodies Olfm4 (Rabbit, CST, 1:1000), β-catenin (Rabbit, CST, 1:1000) and β-actin (Rabbit, CST, 1:5000) for 12 h at 4 °C. Secondary antibodies were subsequently incubated for 1 h at 25 °C before the development of the blots using the Odyssey Infrared Imaging (ChemiDoc MP Imaging System, Bio-Rad, America), and the acquisition software is Image Lab 3.0. The resolution for exported images is 300 DPI. Original western blots for all relevant figures are shown in “Additional file 1: Original uncropped blots of the WB figure in the combined picture”.

### Statistical analyses

Values, presented as means ± SEM or means ± SD, were analyzed statistically using the SPSS 22.0 software. The independent *t*-test was used to compare 2 groups. Data of 3 or more groups were analyzed by one-way ANOVA followed by Duncan multiple comparisons. The number of mice and enteroids in the experiments was indicated in each figure legend. *P < 0.05 and **P < 0.01 were considered statistically significant. All data are representative of at least 3 independent experiments.

## Results

### Early weaning induces small intestinal growth retardation and morphology damage

**Fig. 1:**
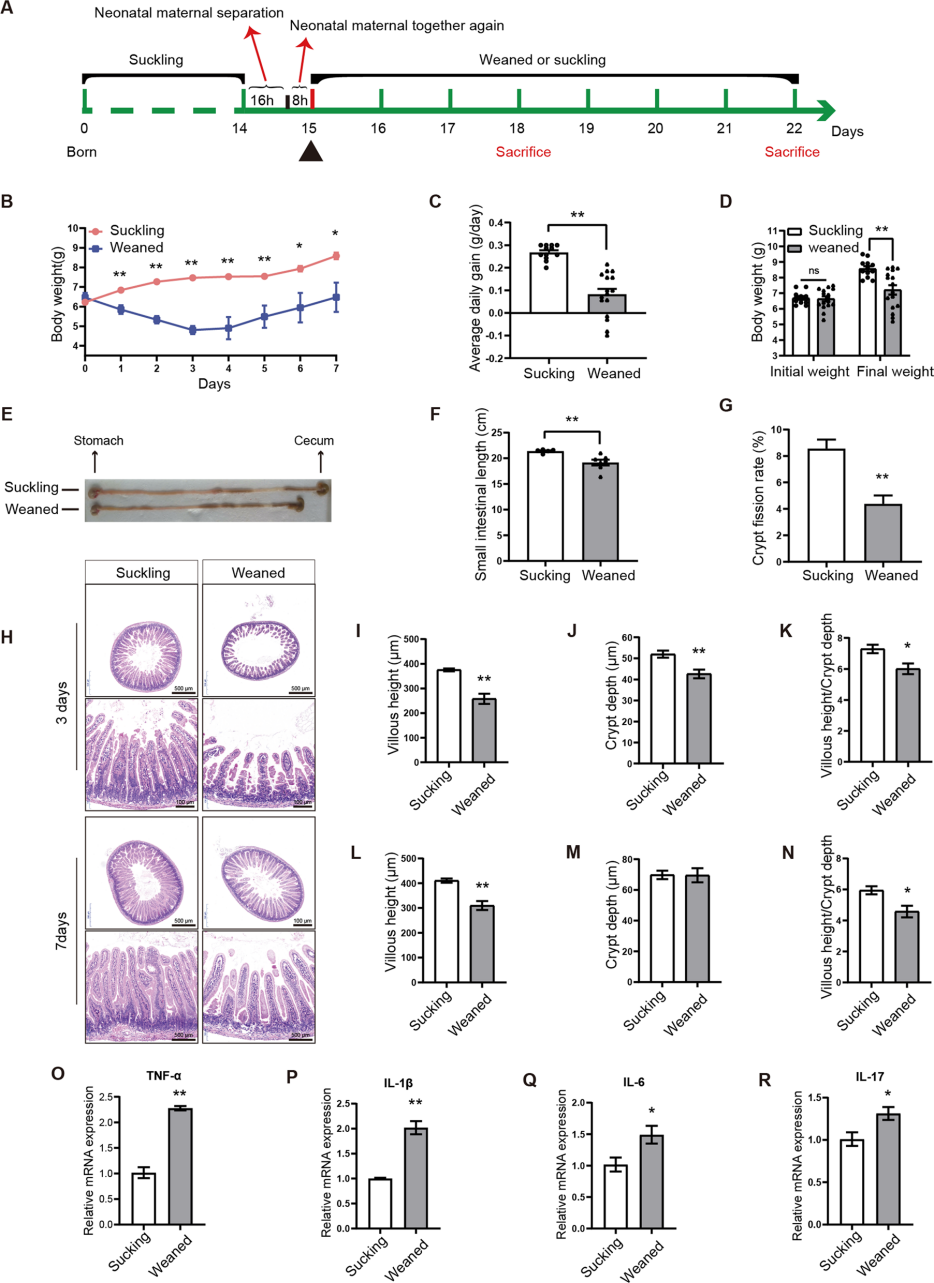
Early weaning induces growth retardation and damage to intestinal morphology. **a** Experimental procedure. **b** The average weight was monitored during the experiment (n = 4–5, means ± SEM, *P < 0.05, **P < 0.01, T-test). **c, d** The average daily gain (**c**), initial weight and final weight (**d**) were measured during the experiment (n = 12 to 16, means ± SEM, *P < 0.05, **P < 0.01, T-test). **e** Representative images of small intestine in suckling mice and early weaning mice on day 7 post-weaning are shown. **f, g** The small intestinal length (**f**) and crypt fission rate (**g**) were determined in mice (n = 5–6, means ± SEM, *P < 0.05, **P < 0.01, T-test). **h** Representative images of H&E staining of jejunum in suckling mice and weaned mice on days 3 and 7 post-weaning are shown. Images were taken using a microscope (Olympus, BX51, Japan) and acquired with OLYMPUS Stream. (J-K) The villous height, crypt depth, and the ratio of villous height to crypt depth were measured in suckling mice and weaned mice on days 3 post-weaning (n = 8, means ± SEM, *P < 0.05, **P < 0.01, T-test). (L-M) The villous height, crypt depth, and the ratio of villous height to crypt depth were measured in suckling mice and early weaning mice on day 7 post-weaning (n = 8, means ± SEM, *P < 0.05, **P < 0.01, T-test). (O-R) RT-qPCR analyses for the expression of proinflammatory in jejunum (n = 4, means ± SEM, *P < 0.05, **P < 0.01, T-test). All the sole presented micrograph figure represents one single microscope field. The whole combined picture was arranged by Adobe Illustrator CC 2015 and Adobe Photoshop CC 2017

The pups of mice in the same litter were weaned at the age of 15-day-old and randomly divided into two groups: the weaned group and the suckling group. The experimental design is shown in Fig. 1a. Body weights were daily monitored during the experiment. The total food consumption by the early weaning group (12 weaning pups in the group, 4 pups * 3 experimental replicates) is 53.8 g (average 0.64 g/d*pups feed intake). Early weaning led to growth retardation in the first week (Fig. 1b). Compared to the suckling group, the average daily gain (Fig. 1c), final weight (Fig. 1d), small intestinal length, and crypt fission rate (Fig. 1e–g) in the weaned group decreased significantly. Remarkable morphological damage in the jejunum was observed in the weaned group (Fig. 1h). The results of jejunum HE staining showed that villous height, crypt depth, and the ratio of villous height to crypt depth significantly decreased on day 3 post-weaning (Fig. 1i–k). Villous height and the ratio of villous height to crypt depth were significantly reduced on day 7 post-weaning (Fig. 1l, n), but there is no significant difference in crypt depth (Fig. 1m). RT-qPCR results showed that early weaning significantly upregulated the mRNA level of proinflammatory cytokines TNF-α, IL-1β, IL-6, and IL-17 in the jejunum (Fig. 1o–r). Collectively, we established an early weaning mice model with small intestinal growth retardation and epithelial atrophy.

### Early weaning damages the proliferation and number of ISCs in vivo

**Fig. 2:**
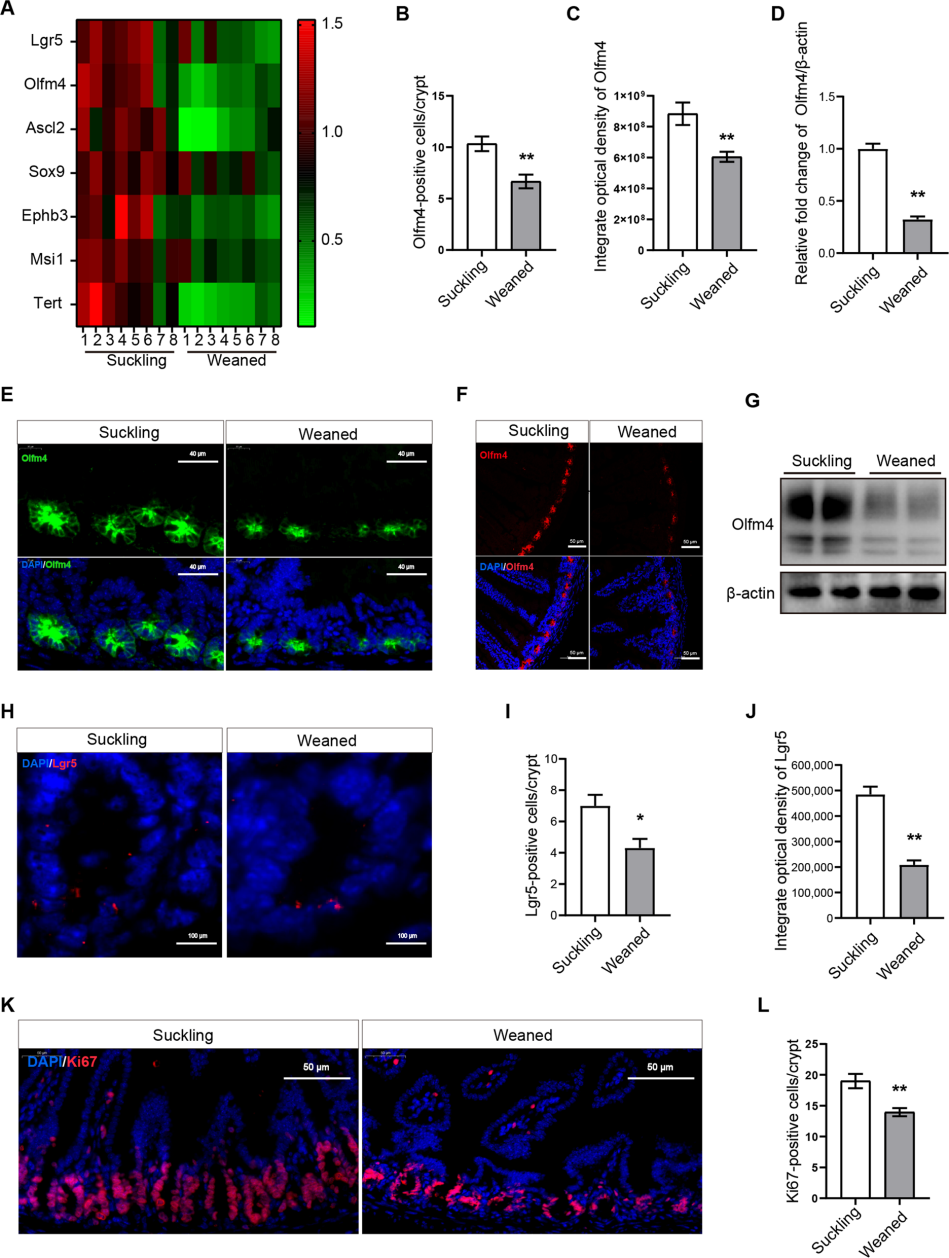
Early weaning inhibits the proliferation and number of ISCs in vivo. **a** Heat maps were generated based on the expression of the significantly changed marker genes of ISCs in vivo. The data represented fold-change differences relative to the suckling group. Genes with a corresponding adjusted *P* value less than 0.05 were considered statistically significant (n = 8, *P < 0.05, T-test). **b, c** Quantification of Olfm4 integrate optical density (**b**) and the number of Olfm4-positive cells (**c**) (n = 6, means ± SEM, *P < 0.05, **P < 0.01, T-test). **d** Quantitative results of immunoblot of Olfm4 (n = 4, means ± SEM, *P < 0.05, **P < 0.01, T-test). **e** ISCs were labeled with Olfm4 (green) in the section of the proximal jejunum. **f** Representative images of ISCs immunofluorescence stained with Olfm4 (red) and DAPI (blue) are shown in the section of the proximal jejunum. **g** Western blot analysis of ISCs marker Olfm4 protein expression in the jejunum. The original uncropped blots were placed in Additional file 1: Fig. 2G. **h** ISCs were labeled with Lgr5 (red) in the section of the proximal jejunum. **i, j** Quantification of Lgr5 integrate optical density and the number of Lgr5-positive cells (n = 4, means ± SEM, *P < 0.05, **P < 0.01, T-test). **k** Representative images of crypt base columnar cells of immunofluorescence stained with Ki67 (red) and DAPI (blue). **l** Quantification of Ki67-positive crypt base columnar cells (n = 6, means ± SEM, *P < 0.05, **P < 0.01, T-test). Jejunal samples collected on day 3 post-weaning were used for the analysis. Immunofluorescence images were taken using a confocal laser scanning microscope (Zeiss, LSM880, Germany) and acquired with ZEN. All the sole presented micrograph figure represents one single microscope field. The whole combined picture was arranged by Adobe Illustrator CC 2015 and Adobe Photoshop CC 2017

Given that considerable growth retardation and intestinal atrophy can be observed on day 3 post-weaning, we collected jejunum samples on this day to investigate the effects of early weaning on ISCs’ homeostasis. We first tested the effect of early weaning on the mRNA levels of ISCs marker genes in jejunum by using RT-qPCR. The mRNA levels of ISCs marker genes *Lgr5, Olfm4, Ascl2, Sox9, Ephb3, Msi1*, and *Tert* were significantly reduced in the weaned group (Fig. 2a). To address how early weaning influences the number of ISCs, we performed immunofluorescence for Olfactomedin-4 (Olfm4, a protein co-expressed with Lgr5) (Fig. 2e, f) and ISCs markers Lgr5 (Fig. 2h) [27, 28]. Early weaning mice had a 36% decrease in the number of Olfm4-positive ISCs (Fig. 2b) and a 38% decrease in the number of Lgr5-positive ISCs (Fig. 2i) compared to the suckling group. Next, we analyzed integrated optical density for Olfm4 and Lgr5, which indicated that early weaning significantly reduced the Olfm4 and Lgr5 protein expression (Fig. 2c, j). Moreover, Western blot results showed that early weaning reduced the expression of Olfm4 at the protein level (Fig. 2d, g). To assess the effect of early weaning on ISCs proliferation, we performed immunofluorescence for Ki67. Compared to suckling group mice, there was a decrease in Ki67 positive crypt base columnar cells, which are ISCs wedged between Paneth cells, indicating that proliferation of ISCs was lower in weaned group mice (Fig. 2k, l). These results suggest that early weaning depresses ISC’s self-renewal activity.

### Early weaning depresses the activity of ISCs to drive epithelial regeneration and crypt expansion in ex-vivo

**Fig. 3:**
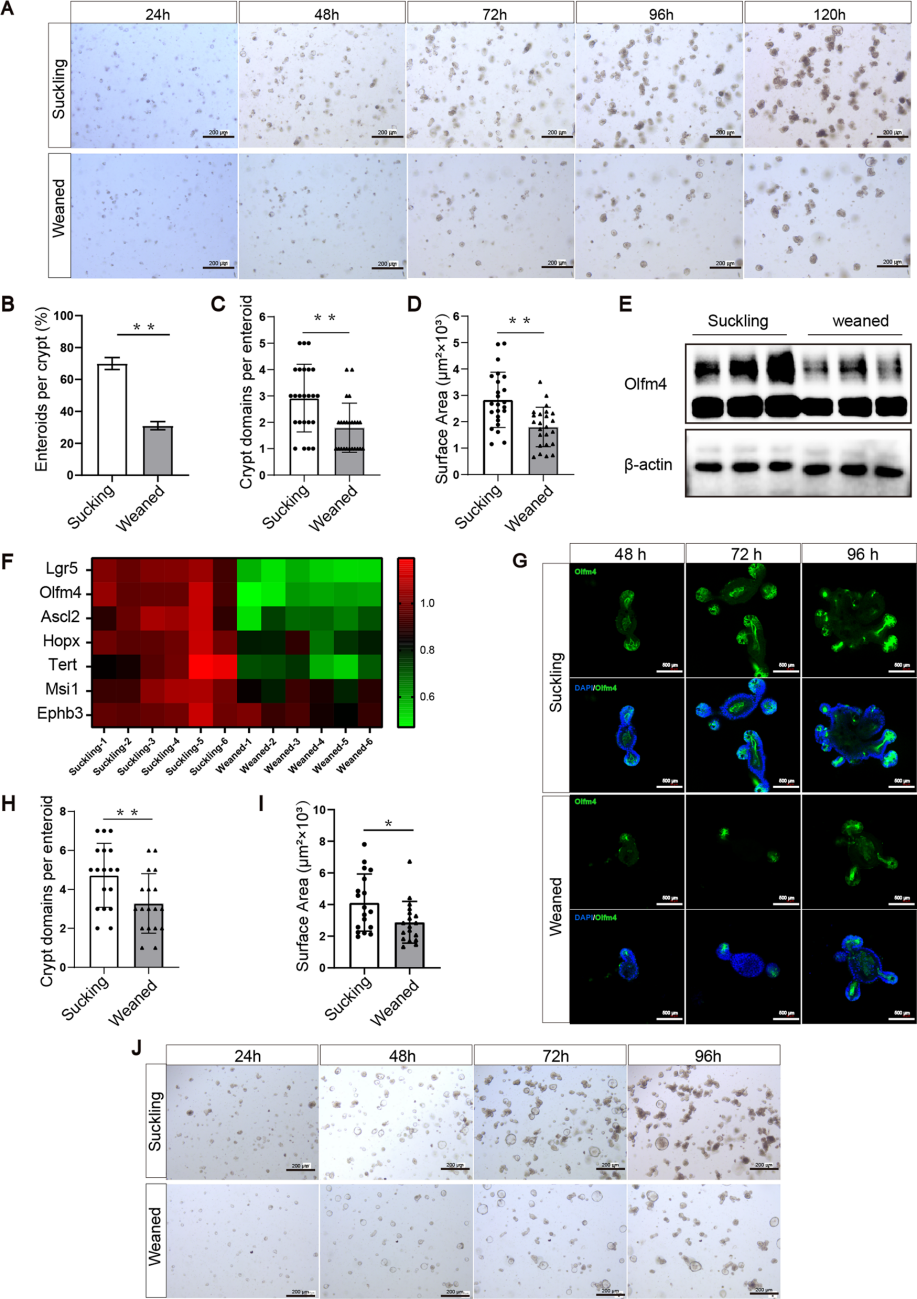
Early weaning attenuates stemness of ISCs in ex-vivo. **a** Representative images of the morphology of primary enteroids expanded from crypt cells at 24 h, 48 h, 72 h, 96 h, and 120 h are shown. Images were taken using a microscope (Nikon, TE2000, Japan) and acquired with NIS-Elements. **b** Enteroid-forming capacity of mice jejunum crypts (n = 4, means ± SD, *P < 0.05, **P < 0.01, T-test). **c, d** Quantification of crypt domains (**c**) and surface area (**d**) of per primary enteroid at 96 h (n = 4, means ± SD, *P < 0.05, **P < 0.01, T-test). **e** Western blot analysis of ISCs marker Olfm4 protein expression in primary enteroids. The original uncropped blots were placed in Additional file 1: Fig. 3E. **f** Heat maps were generated based on the expression of the significantly changed marker genes of ISCs in primary enteroids. The data represented fold-change differences relative to the suckling group. Genes with a corresponding adjusted *P* value less than 0.05 were considered statistically significant (n = 6 *P < 0.05, T-test). **g** Representative images of primary enteroids of immunofluorescence stained with Olfm4 (green) and DAPI (blue) are shown. **h, i** Quantification of crypt domains (**h**) and surface area (**i**) of per secondary enteroids (n = 3, means ± SD, *P < 0.05, **P < 0.01, T-test). Immunofluorescence images were taken using a confocal laser scanning microscope (Zeiss, LSM880, Germany) and acquired with ZEN. **j** Representative images of the morphology of secondary enteroids expanded from crypt cells at 24 h, 48 h, 72 h, and 96 h are shown. All the sole presented micrograph figure represents one single microscope field. The whole combined picture was arranged by Adobe Illustrator CC 2015 and Adobe Photoshop CC 2017

Because early weaning decreases the self-renewal of ISCs and causes intestinal epithelial atrophy in vivo, we ask whether early weaning also affects the activity of ISCs to drive intestinal epithelial regeneration and crypt expansion in ex-vitro. We assessed the enteroid-forming ability of isolated jejunum crypts, which is a proxy for ISC function [19, 29]. We found that early weaning significantly inhibited the enteroid-forming capacity of jejunum crypts, reduced enteroids size, and crypt domain structure (Fig. 3a–d). Compared to suckling group, early weaning reduced the number of Olfm4-positive ISCs in primary enteroids crypts (Fig. 3g), which is consistent with in vivo data shown in Fig. 2b, e. Furthermore, the RT-qPCR results showed a lower mRNA level of ISCs-relative markers in primary enteroids from the weaned group (Fig. 3f), which is consistent with in vivo data (Fig. 2a). Western blot results showed that early weaning reduced the protein level of Olfm4 in primary enteroids (Fig. 3e). Interestingly, when sub-cloned, secondary enteroids from the weaned group had fewer crypt domains and smaller size of enteroid than the suckling group (Fig. 3h–j). Taken together, these results suggest that early weaning depresses the activity of ISCs-driven epithelial regeneration and crypt expansion.

### Early weaning attenuates Wnt/β-catenin signaling in ISCs in vivo and ex-vivo

**Fig. 4:**
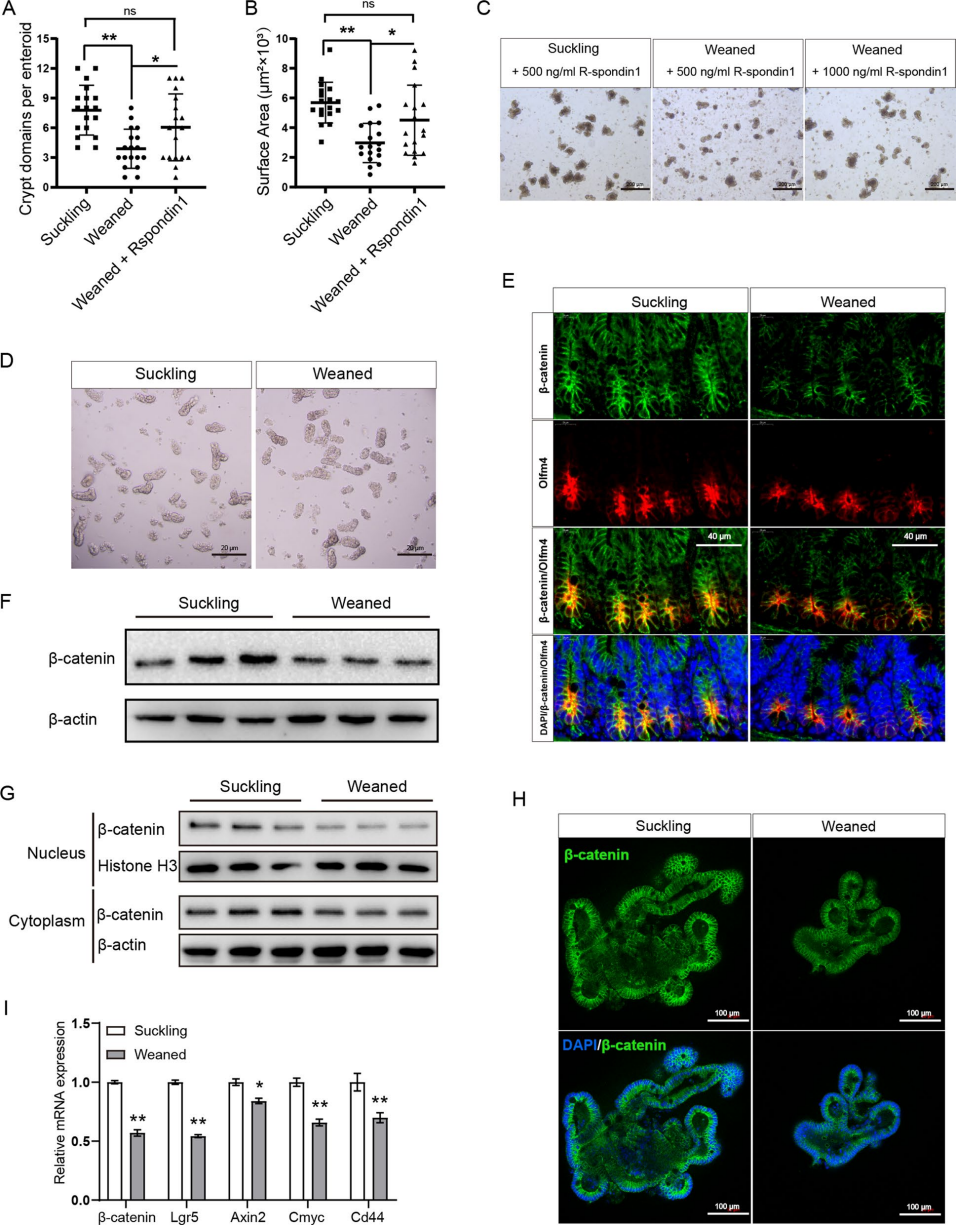
Early weaning inhibits Wnt signaling in ISCs in ex-vivo and in vivo. **a, b** Inhibitory effect of weaning stress on enteroids crypt domain (**a**) and surface area (**b**) were rescued by exogenous Wnt signaling amplifier R-spondin1 (n = 3, means ± SD, *P < 0.05, **P < 0.01, one-way ANOVA). **c** Representative images of primary enteroids at 96 h are shown. **d** Representative images of crypts isolated from jejunum were shown. Images were taken using a microscope (Nikon, TE2000, Japan) and acquired with NIS-Elements. **e** Representative images of jejunum crypts immunofluorescence stained with β-catenin (green), Olfm4 (red), and DAPI (blue). **f** Western blot analysis β-catenin protein expression in jejunum crypts. The original uncropped blots were placed in Additional file 1: Fig. 4F. **g** Levels of β-catenin crypt domains of primary enteroids crypt domains in cytoplasm and nucleus were measured by Western blot at 96 h. The original uncropped blots were placed in Additional file 1: Fig. 4G. **h** Representative images of primary enteroids of immunofluorescence stained with β-catenin (green) and DAPI (blue). Immunofluorescence images were taken using a confocal laser scanning microscope (Zeiss, LSM880, Germany) and acquired with ZEN. **i** RT-qPCR analyses for the expression of multiple Wnt-target genes in primary enteroids crypt domains (n = 6, means ± SEM, *P < 0.05, **P < 0.01, T-test). All the sole presented micrograph figure represents one single microscope field. The whole combined picture was arranged by Adobe Illustrator CC 2015 and Adobe Photoshop CC 2017

The Wnt/β-catenin pathway is critical for ISCs’ function, which prompts us to hypothesize that early weaning attenuates the function of ISCs through Wnt signaling. First, we tested whether increasing the content of exogenous signaling amplifier R-spondin1 in the culture medium can rescue early weaning-induced suppression of ISCs function. We found that the exogenous Wnt amplifier supplementation with a double dose of the control group reversed the effects of early weaning on crypt domains and surface areas in newly initiated primary enteroids (Fig. 4a–c). Next, given that ISCs are at the bottom of the crypt, we separated crypts from jejunum in mice to examine the expression of β-catenin, a surrogate for canonical Wnt signaling (Fig. 4d). Western blot results showed that the expression of β-catenin was reduced in jejunum crypts from weaned group mice (Fig. 4f). Immunofluorescence results also showed that early weaning reduced the expression of β-catenin in ISCs of jejunum crypts (Fig. 4e). Lastly, we tested the expression of β-catenin in primary crypt domains of primary enteroids, which indicated that early weaning reduced the expression of β-catenin (Fig. 4g, h). Western blot results also showed less nuclear β-catenin in crypt domains of primary enteroids from weaned group compared to the suckling group (Fig. 4g). Additionally, early weaning reduced the mRNA levels of Wnt-responsive genes, such as *Axin2, Cmyc, Cd44, β-catenin, and Lgr5* in primary enteroids crypt domains (Fig. 4i). To summarize, these results suggest that early weaning attenuates ISCs function by decreasing Wnt signaling.

### Early weaning hinders small intestinal epithelial regeneration and affects the differentiation of ISCs

**Fig. 5:**
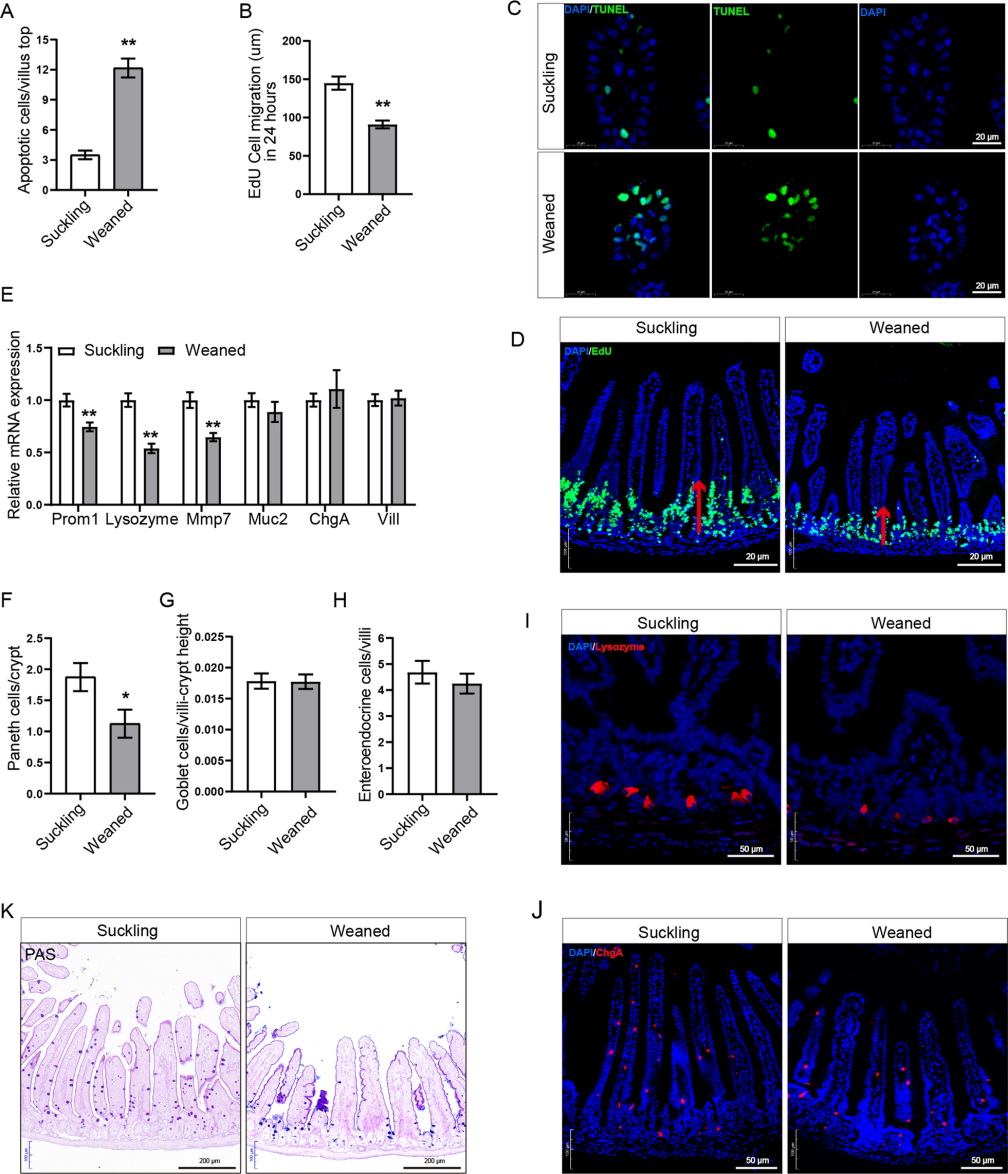
Effects of early weaning on ISCs differentiation and epithelial regeneration in vivo. **a** Quantification of apoptotic cells per villus top (n = 6, means ± SEM, *P < 0.05, **P < 0.01, T-test). **b** Quantification of cell migration height after 24 h EdU Labeling. (n = 4, means ± SEM, **P < 0.01, T-test). **c** Representative images of immunofluorescence represent the overlap of positive signal (green) and nuclear signal (blue). **d** Representative confocal images of cell migration in suckling and weaning mice with 24 h EdU labeling. **e** Relative mRNA expressions of Transit-amplifying cells marker *prom1*, Paneth cells markers *Lysozyme* and *Mmp7*, goblet cells marker *Muc2*, enteroendocrine cells *ChgA*, and enterocytes marker *vill* in vivo were analyzed by RT-qPCR (n = 6, means ± SEM, *P < 0.05, **P < 0.01, T-test). **f–h** The number of Paneth cells (**f**), goblet cells (**g**), and enteroendocrine cells (**h**) per crypt-villus were quantified (n = 6, means ± SEM, *P < 0.05, T-test). **i, j** Representative images of immunofluorescence stained with Lysozyme (red), ChgA (red), and DAPI (blue) in the jejunum. Immunofluorescence images were taken using a confocal laser scanning microscope (Zeiss, LSM880, Germany) and acquired with ZEN. **k** Representative images of goblet cells. Images were taken using a microscope (Olympus, BX51, Japan) and acquired with OLYMPUS Stream. All the sole presented micrograph figure represents one single microscope field. The whole combined picture was arranged by Adobe Illustrator CC 2015 and Adobe Photoshop CC 2017

To figure out the mechanism of early weaning-induced intestinal epithelial atrophy, we first carried out a TUNEL experiment to evaluate the number of apoptotic epithelial cells in small intestinal villi tops. As we expected, early weaning increased the number of apoptotic cells at the top of small intestinal villi (Fig. 5a, c). Next, to assess the rate of intestinal epithelial renewal, we measured the migration height of proliferating and differentiating cells from the crypt to the villus compartment, after EdU^+^ labeling for 24 h. We found that the migration height of EdU-positive cells in the weaned group was 37% lower than that in the suckling group (Fig. 5b, d). To study the effects of early weaning on the differentiation of ISCs, we collected samples of jejunum in mice on day 3 post-weaning and extracted total RNA from the jejunum tissue. RT-qPCR was performed to study the mRNA levels of differentiation markers in the jejunum on day 3 post-weaning. RT-qPCR results showed that early weaning significantly reduced the mRNA level of transit-amplifying cells marker *prom1* (Fig. 5e). There are no differences in the expression of enterocyte cells marker *Vill*, goblet cells marker *Muc2*, and enteroendocrine cells marker *ChgA* between the weaned group and the suckling group (Fig. 5e). Immunofluorescence results show that early weaning did not change the number of endocrine cells in the small intestinal epithelium (Fig. 5h, j), which is consistent with RT-qPCR results. PAS results show that early weaning also did not change the number of goblet cells (Fig. 5g, k), which is consistent with RT-qPCR results. In contrast, the weaned group had significantly lower mRNA levels of Paneth cell markers *lysozyme* and *Mmp7* than that in the suckling group (Fig. 5e). Consistent with RT-qPCR results, early weaning reduced the number of Paneth cells in vivo (Fig. 5f, i). In summary, early weaning increases the apoptosis of small intestinal epithelial cells, hinders small intestinal epithelial regeneration, and affects the differentiation of ISCs.

## Discussion

Early weaning and shorter breastfeeding duration are applied by a proportion of young mothers, especially in the social spheres of poverty-stricken areas. This early weaning practice was revealed to increase the incidence of gastrointestinal disease and cause intestinal atrophy in mammals. Early childhood is a critical period for intestinal development, which is driven by intestinal stem cells (ISCs). Therefore, elucidating the effects of early weaning on the ISCs’ activity may provide new ideas for the prevention and treatment of early weaning-induced intestinal problems from the perspective of ISCs. Interestingly, in the early weaning mice model, we found that early weaning practice depresses ISCs’ function via attenuating Wnt signaling in vivo and ex vivo, which suggests that increasing Wnt signaling in ISCs during early weaning may be an effective way to alleviate early weaning-induced intestinal problems.

In the swine production industry, piglets are commonly weaned at age of 3 or 4 weeks [30], which may serve as a natural early weaning model for investigating the response of ISCs to early weaning. However, the lack of effective antibodies for the porcine, such as Lgr5 and Olfm4, increases the difficulty to identify ISCs (columnar base cells in the crypt) in the pig models [31, 32]. In addition, the lack of standardized porcine enteroids culture methods is also an obstacle to investigating porcine ISCs in ex-vivo and in vitro [33–35]. Instead, with the development of validated reagents and enteroids culture methods for mice, mice enteroid is an available and acceptable model for studying intestinal development and diseases [36]. Therefore, for the first time, we established an early weaning mice model and the intestinal organoids from mice to explore how ISCs in mammals respond to early weaning. In the novel model, early weaning mice developed symptoms including a marked decrease in villous height, a significant decline in small intestine length, a reduced growth rate of body weight, and upregulation of proinflammatory cytokines, which are similar to the early weaning piglets during the first week of weaning [37, 38]. Overall, we establish a readily accessible mice model that effectively recapitulates key features of intestinal atrophy induced by early weaning. Previous studies have shown that early life stress imposes detrimental impacts on intestinal epithelial homeostasis and increases risks for functional gastrointestinal disorders, such as irritable bowel syndrome (IBS) in later life [39, 40]. Whether the early weaning mouse model established in our study will exhibit adverse effects on intestinal health in later life needs to be further observed.

ISCs’ self-renewal homeostasis, including number and proliferation, is easily disrupted in response to challenges from the luminal environment. For example, rotavirus infection will increase ISCs’ number and proliferation, which helps accelerate the regeneration of intestinal epithelium to weed out the infected enterocytes [16]. In contrast, inflammation, endoplasmic reticulum stress, and pathogen infection will weaken or even disable the self-renewal of ISCs, which increases the difficulty of the intestine recovering from the epithelial damages [17, 18, 41, 42]. In the current study, we found that early weaning attenuated the expression of ISCs’ stemness markers in vivo and ex-vivo. Further results verified that early weaning decreased both the number and proliferation of ISCs. Therefore, it is concluded that early weaning depresses ISC’s self-renewal, which suggests that insufficient or overconsumption of ISCs may be involved in early weaning-induced intestinal atrophy.

The small intestinal epithelium is one of the fastest renewing tissues, regenerating every 2–4 days [10, 11]. Moreover, small intestinal crypts have a high fission rate to support the growth of small intestines in infants and young animals [43–45]. Given that small epithelial regeneration and crypt fission are driven by ISCs [23, 46], we tested whether early weaning affects the activity of ISCs to drive epithelial regeneration and crypt fission. We found that early weaning stress decreased the enteroids formation, crypts domains, and enteroids size, which are proxies for ISC activity [19, 29], in primary and secondary enteroids. In vivo results showed that the EdU-positive cell migration and the crypt fission rate in early weaning mice were significantly lower than that in suckling mice. These findings reveal that early weaning stress depressed the activity of ISCs to drive epithelial regeneration and crypt fission, which may explain the mechanism of early weaning-induced small intestinal epithelial atrophy and growth retardation from the perspective of ISCs.

Early weaning induces significant morphological changes in the intestinal epithelium in the piglet model, including shortening of villi (villus atrophy) and a marked decrease in villus: crypt ratio [37]. In our study, we found similar intestinal epithelial morphological damage in early weaning mice. However, the cytologic process involved in this morphological damage during weaning is still incompletely understood [37]. We found that the number of apoptotic enterocytes at the top of villi in the weaned group is 3.47 times higher than that in the suckling group. In contrast, the rate of intestinal epithelial regeneration, reflected by the migration height of EdU-positive cells from the crypt to villus top, in the weaned group was decreased by 37%, which is consistent with the decrease of cell proliferation marker Ki67 in the crypt and transit-amplifying cells marker in the weaned group. These results are similar to the previous findings that proliferating cells in the crypt were decreased in weaning piglets [31]. Consequently, we hypothesize that excessive apoptosis of enterocytes, which shedding from the villus top, and slow migration of regenerated cells from the crypt to the villus top jointly lead to intestinal epithelial morphological damage during weaning. A previous study demonstrated that early life stress changes the proportion of intestinal epithelial cells by affecting the differentiation of ISCs [21]. In the present study, we found that early weaning reduced the number of Panth cells, which may partly explain the weakening of ISCs’ function caused by early weaning because Paneth cells provide essential niche signals for maintaining ISCs’ function [47]. We also found early weaning inhibited the differentiation of ISCs into transit-amplifying cells, which means that intestinal epithelial regeneration was slowed down. Taken together, it can be inferred that both excessive enterocytes’ apoptosis and depressed ISC’s function are involved in intestinal epithelium morphological damage caused by early weaning.

Elucidating the molecular mechanism of the decline in ISCs’ activity caused by early weaning is important to develop new interventions for early weaning-induced intestinal problems. In the cases of intestinal epithelial injury, Wnt signaling plays a crucial role in mediating the homeostasis of ISCs [22, 23]. We, therefore, hypothesized that Wnt signaling may be involved in the weakening of ISCs function during early weaning. In the present study, we found that the exogenous Wnt signaling amplifier reversed the inhibitory effect of early weaning on ISCs’ function. Consistently, early weaning inhibited the expression of ISCs Wnt-responsive genes and decreased the level of ISCs nuclear β-catenin, a surrogate for canonical Wnt signaling [47]. Besides, the decrease in the number of Panth cells observed in the early weaning group may partly explain the weakening of Wnt signaling in ISCs caused by early weaning, because Paneth cells secrete Wnt ligands for ISCs Wnt signaling activation [47]. Therefore, it can be concluded that early weaning depresses ISCs’ function via attenuating Wnt signaling, which suggests that increasing Wnt signaling in ISCs may be an effective way to alleviate early weaning-induced intestinal atrophy.

Although our study showed that early weaning causes small intestinal atrophy by inhibiting the activity of ISCs via suppression of Wnt/β-catenin signaling, it was not clearly determined how early weaning cause suppression of Wnt/β-catenin signaling. Previous studies have shown that T help cell cytokines, such as TFNγ, IL-10, IL-17, and IL-13, modulate ISCs renewal and differentiation and involve the activity of Wnt signaling of ISCs [48–50]. Interestingly, in the present study, we found that weaning stress increased the expression of proinflammatory cytokines TNF-α, IL-1β, IL-6, and IL-17 in jejunum, which may be the potential mechanism participating in the Wnt signaling suppression. The relationship among proinflammatory cytokines, intestinal stem cell activity, and the early weaning practice should be concerned in further research.

## Conclusion

**Fig. 6:**
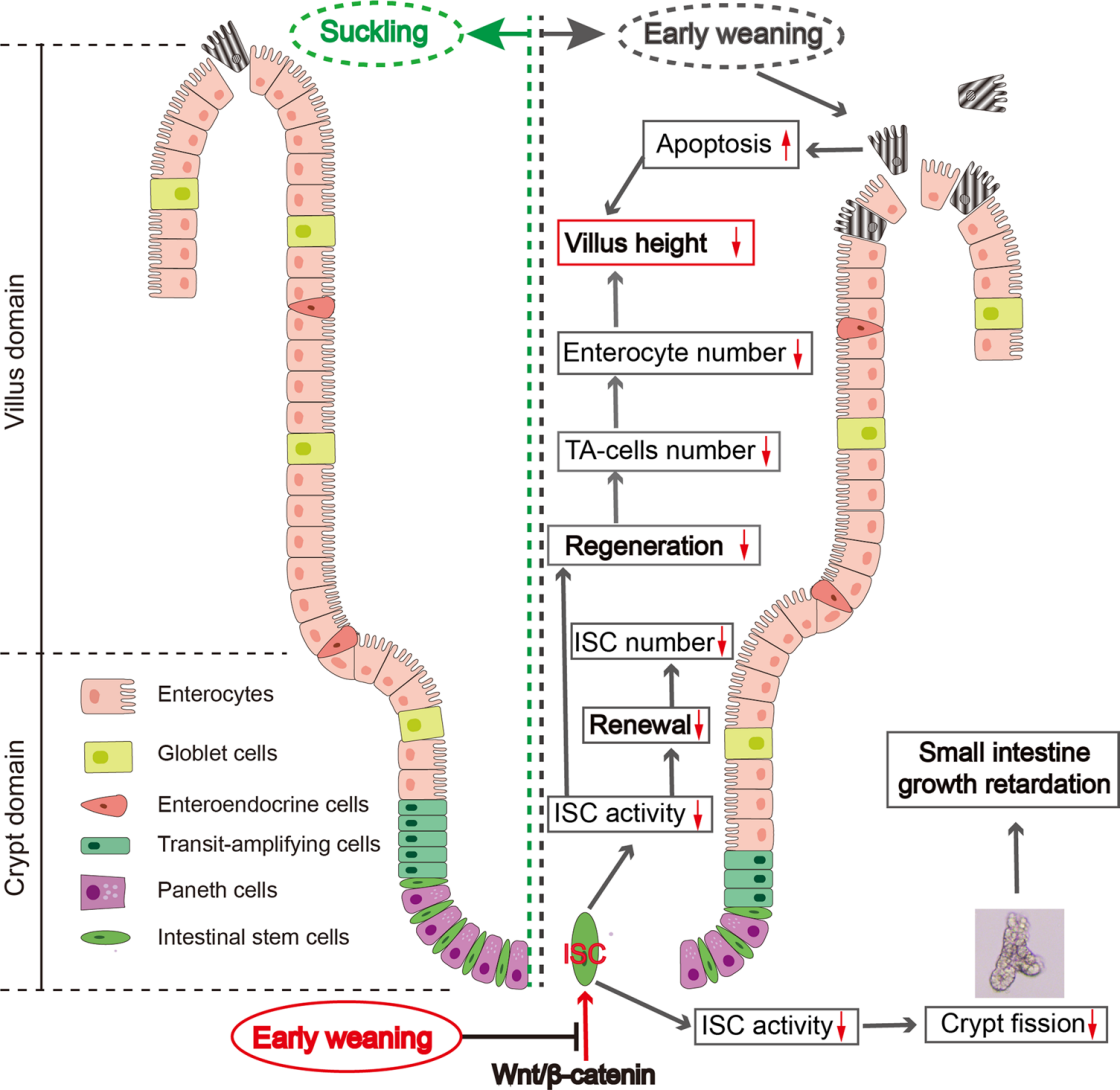
Schematic diagram showing early weaning inhibits the activity of ISCs-driven epithelial regeneration and crypt fission via suppression of Wnt/β-catenin. The image of the crypt depicted in this figure was adopted from our origin, and the schematic diagram was created with Adobe Illustrator CC 2015

In this study, for the first time, we established an excellent early weaning mice model that has significant intestinal atrophy and growth arrest symptoms to explore the response of ISCs to early weaning. Specifically, early weaning depresses the activity of ISCs-driven intestinal epithelial regeneration and crypt fission by attenuating Wnt signaling. Particularly, the triggered proinflammatory cytokines TNF-α, IL-1β, IL-6, and IL-17 in jejunum may be a potential mechanism participating in this Wnt signaling suppression. As a result, the cells regenerated from ISCs fail to timely compensate for excessive apoptotic intestinal epithelial cells and drive crypt fission, resulting in intestinal epithelium atrophy and small intestinal growth retardation (Fig. 6). Our findings provide novel insights into early weaning-induced intestinal epithelial atrophy from the perspective of ISCs, which may guide the development of infant nutrients to prevent and rescue early weaning-induced intestinal problems.

## Supplementary Information

**Additional file 1**. Original uncropped blots of the WB figure in the combined picture.

## Data Availability

The data that support the findings of this study are available on request from the corresponding author. The data are not publicly available due to privacy or ethical restrictions.

## Abbreviations

EdU: 5-Ethynyl-2′-deoxyuridine
EGF: Epidermal growth factor
HE: Hematoxylin and eosin
ISCs: Intestinal stem cells
PBS: Phosphate buffer saline
PVDF: Polyvinylidene difluoride
TUNEL: Terminal deoxynucleotidyl transferase-mediated dUTP nick labeling
WHO: World health organization
