# Supplementary figures and images for: Early weaning causes small intestinal atrophy by inhibiting the activity of intestinal stem cells: involvement of Wnt/β-catenin signaling
Source: Stem Cell Res Ther. 2023 Apr 5;14:65. doi: 10.1186/s13287-023-03293-9 (PMC10077674; doi:10.1186/s13287-023-03293-9)

**Supplementary material-original full-length gels and blot**


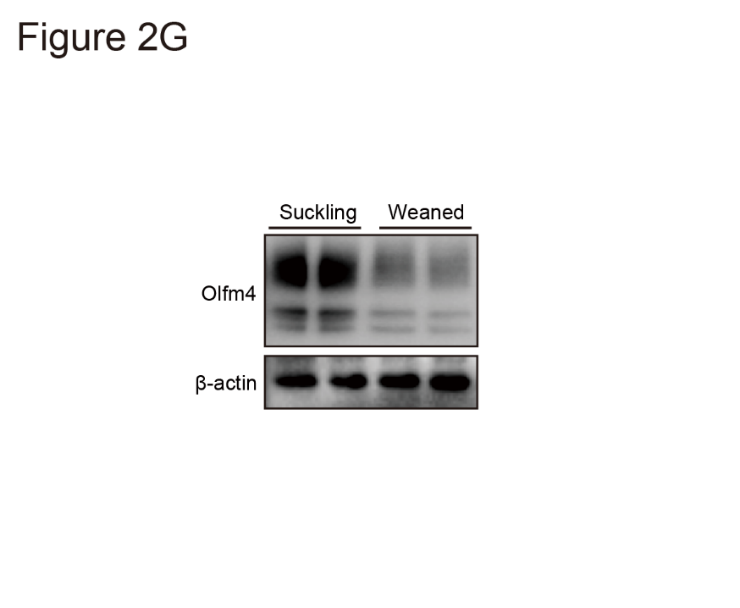


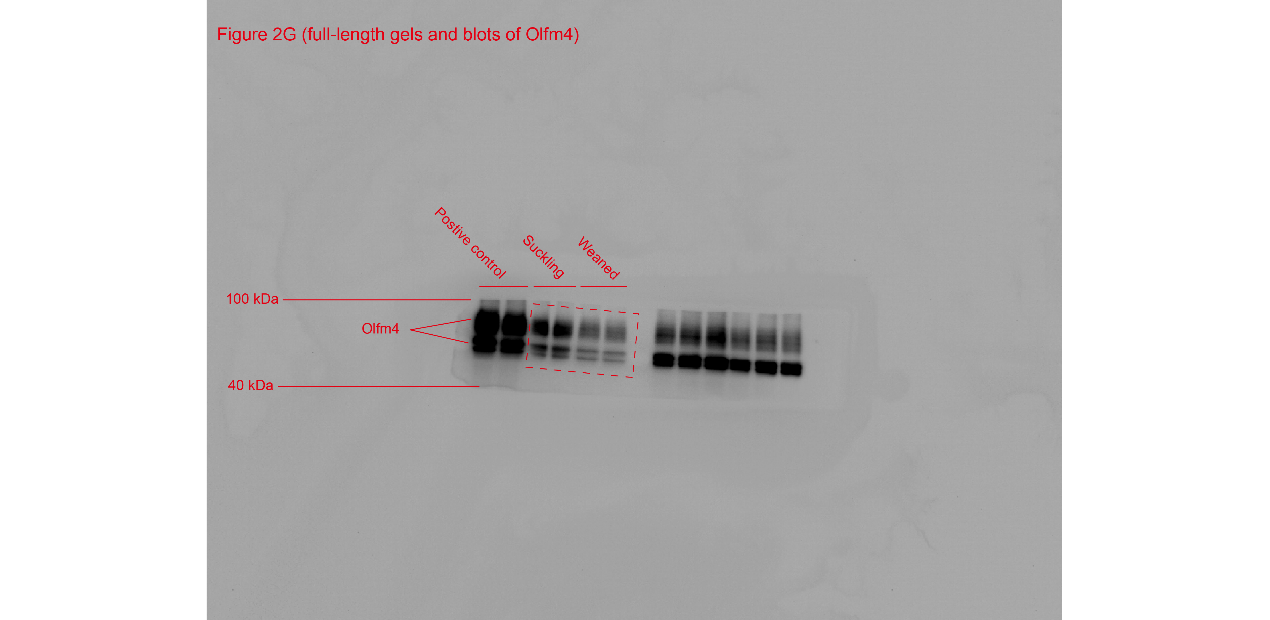


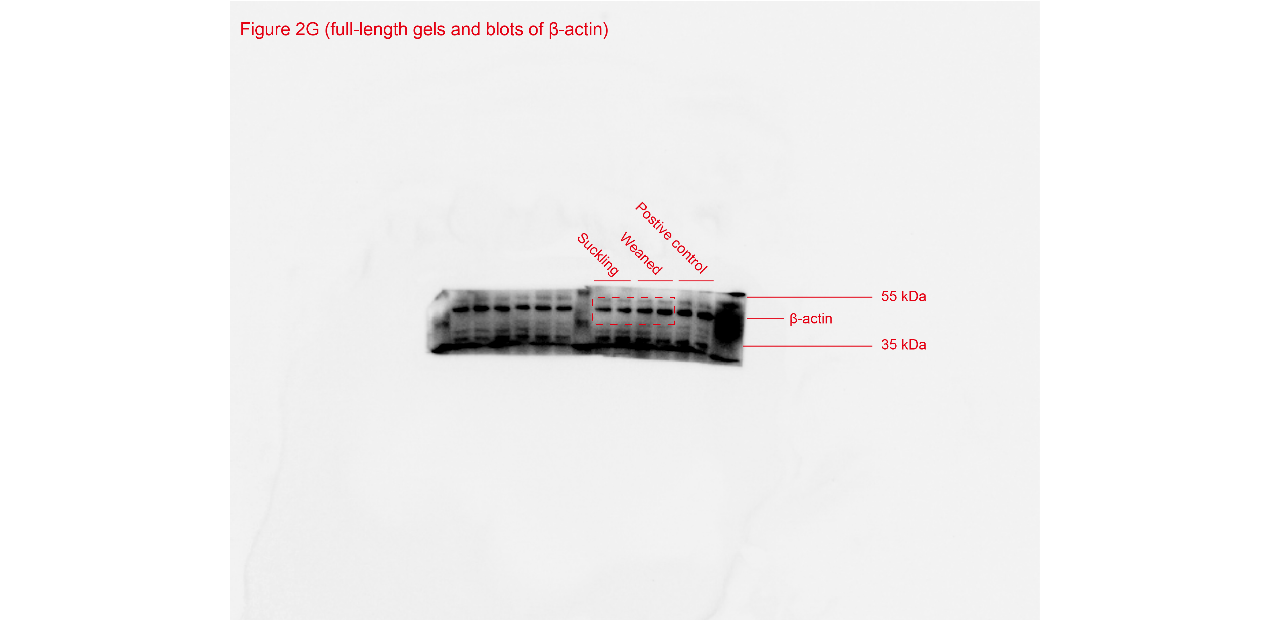


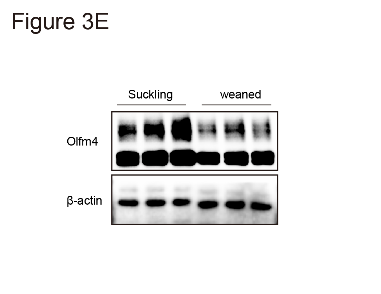


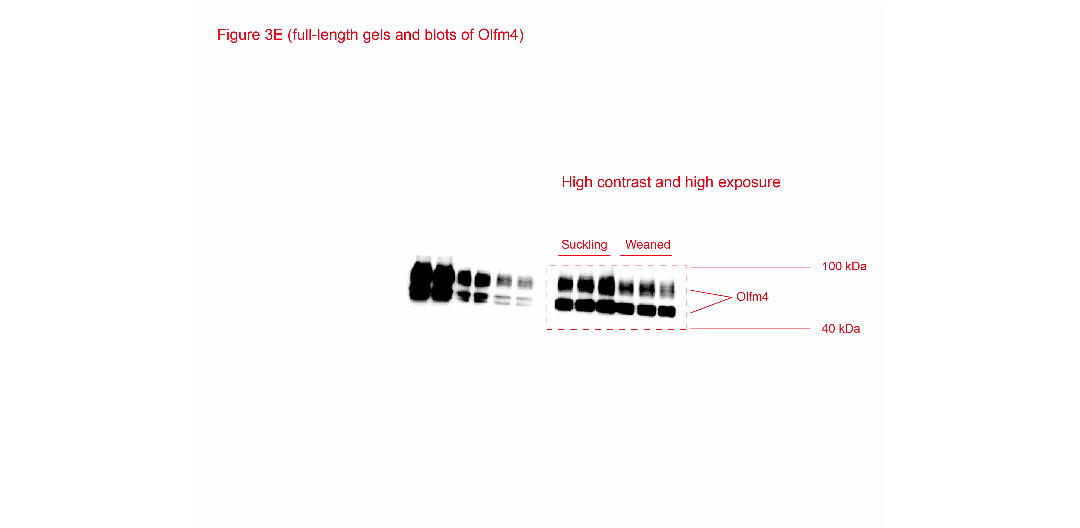


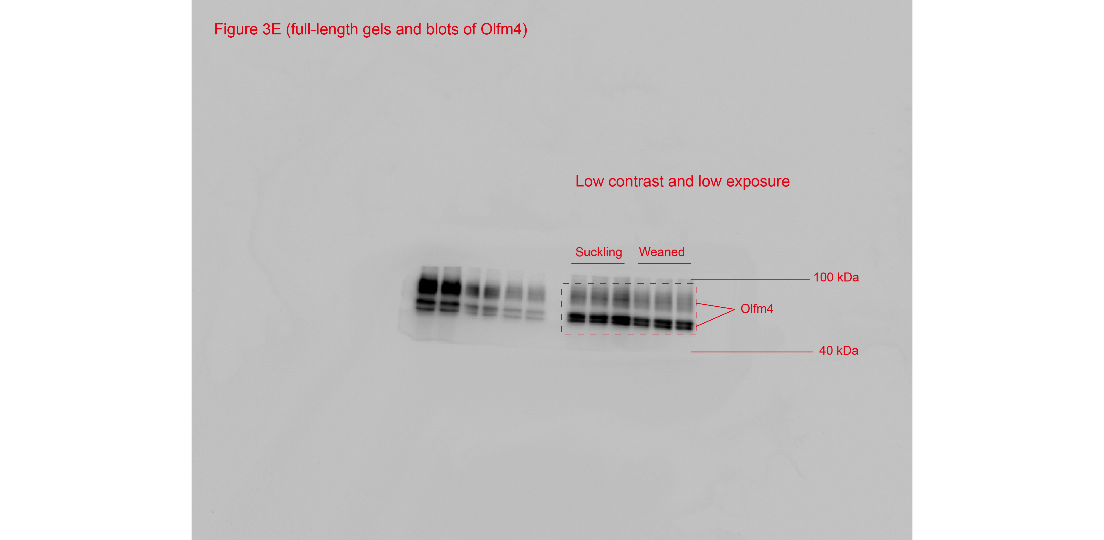


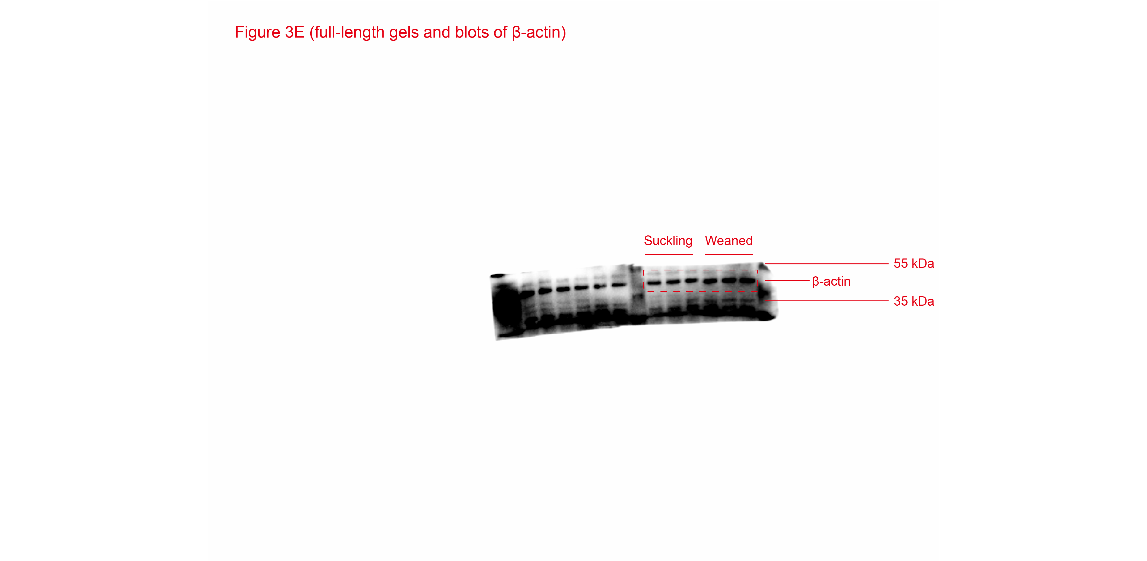


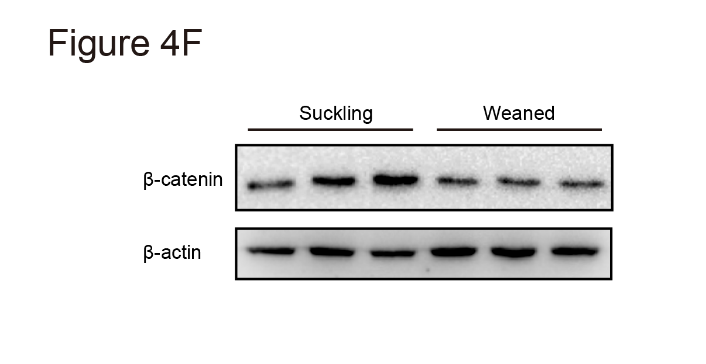


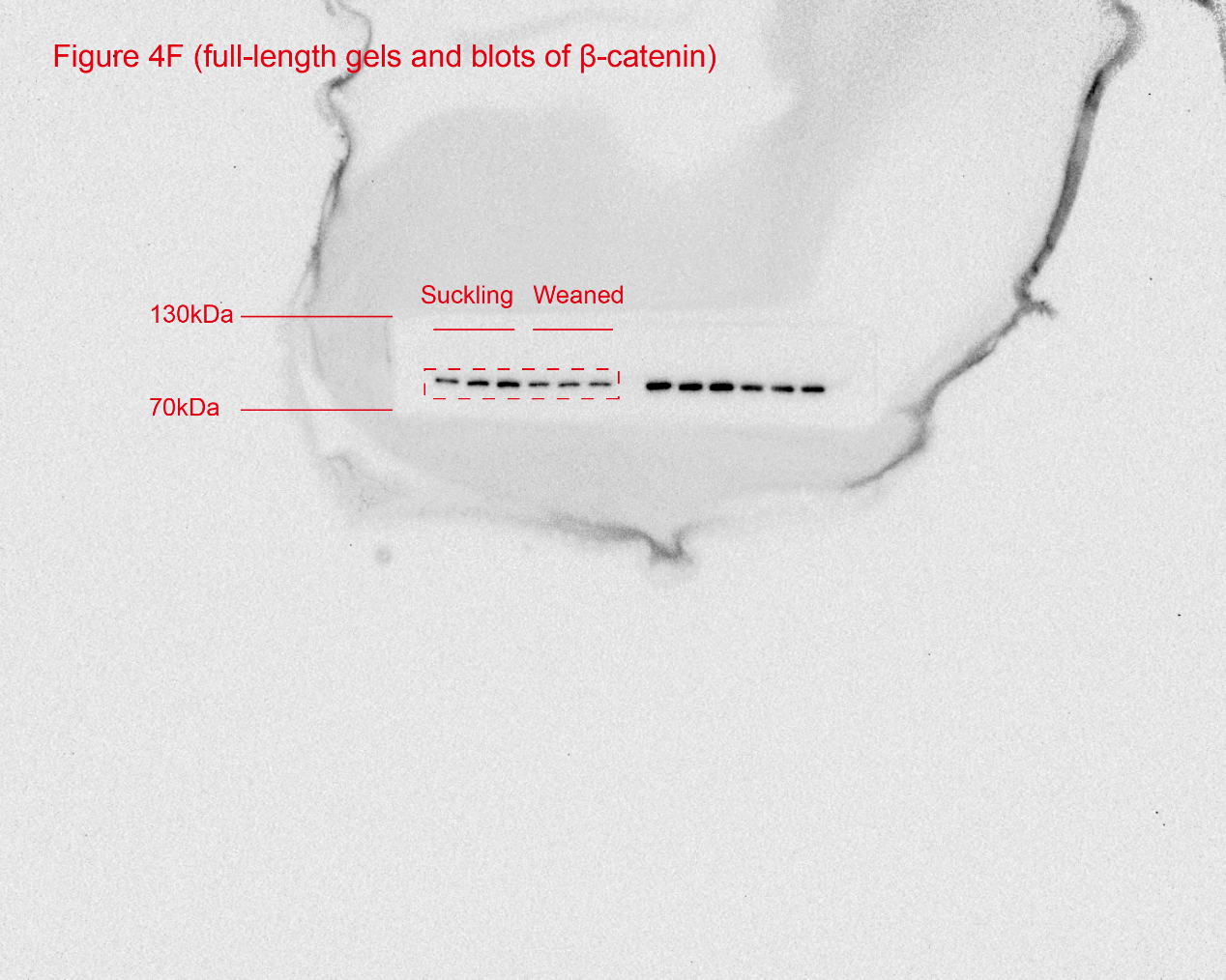


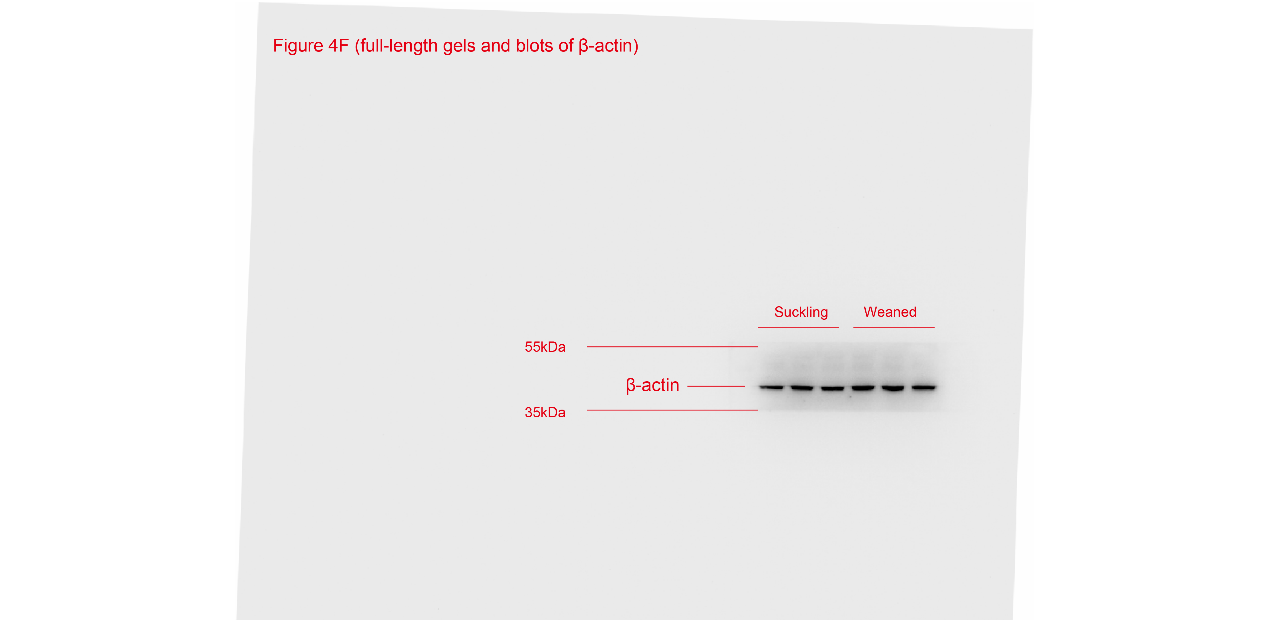


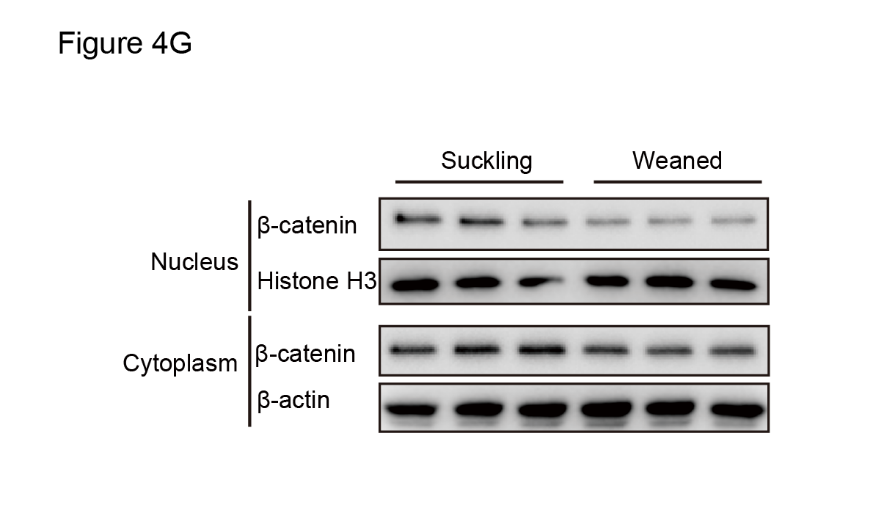


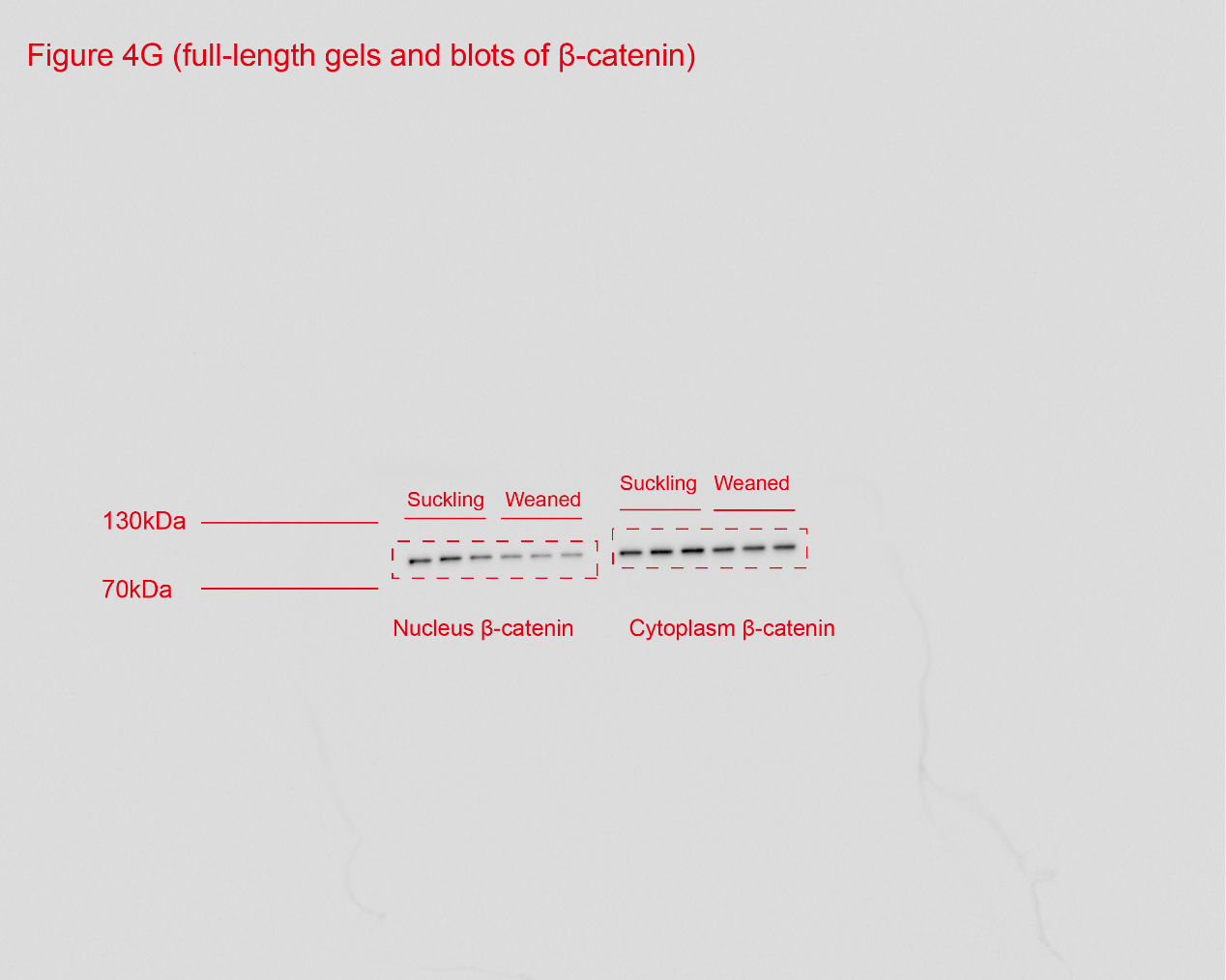


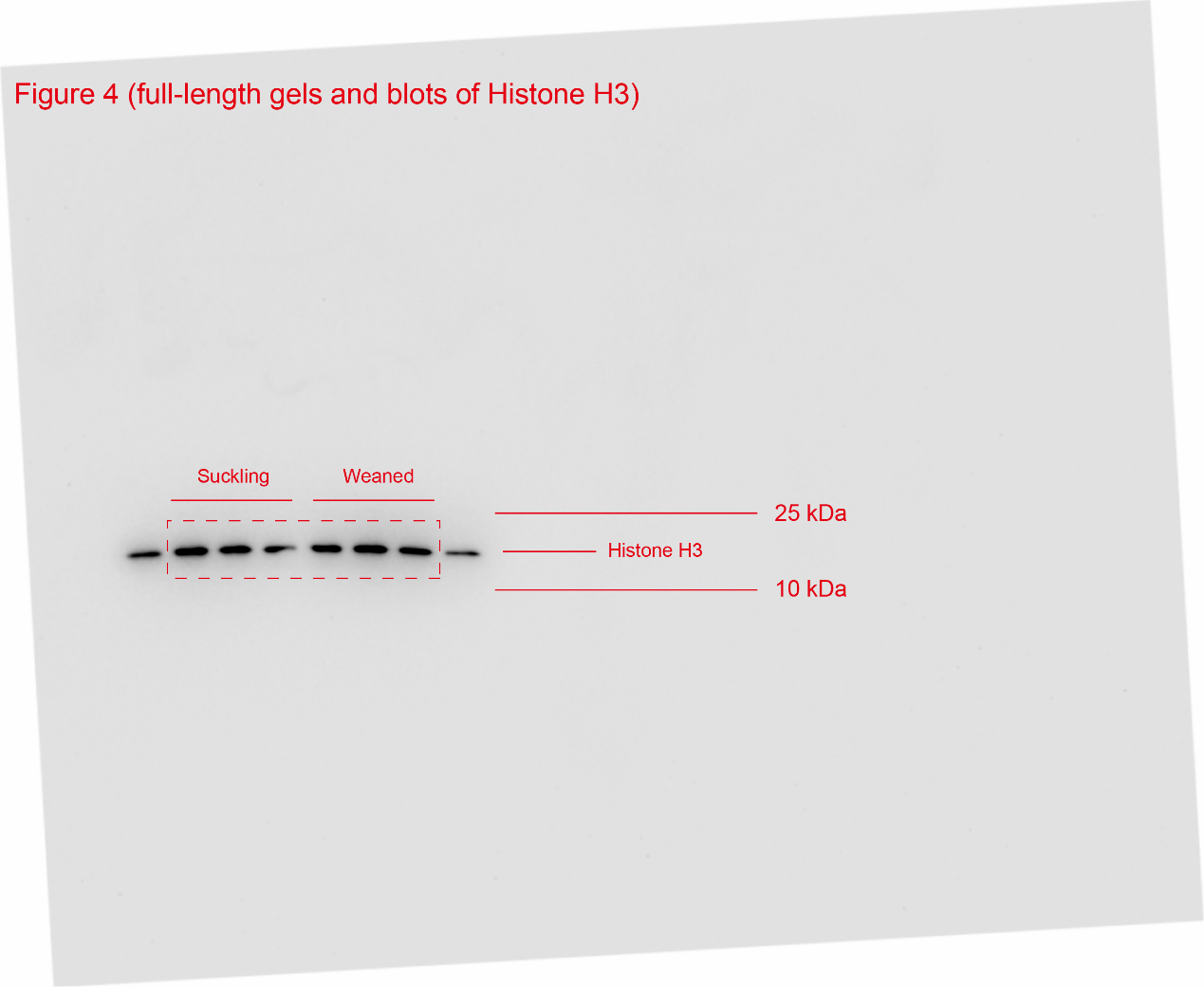


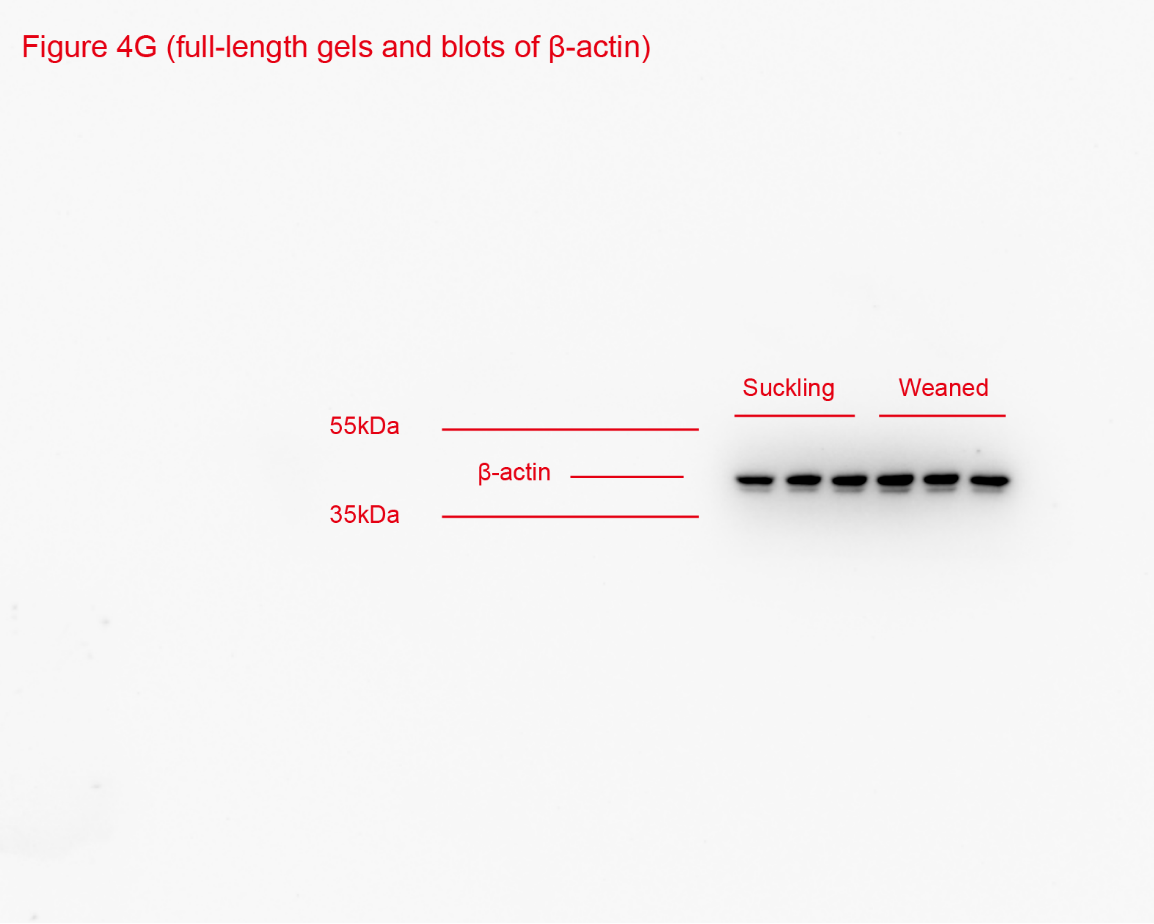

Supplement: Supplementary file 1 — Additional file 1. Original uncropped blots of the WB figure in the combined picture. [file 13287_2023_3293_MOESM1_ESM.docx]
